# Case report: A heterozygous mutation in *ZNF462* leads to growth hormone deficiency

**DOI:** 10.3389/fgene.2022.1015021

**Published:** 2022-12-07

**Authors:** Yikun Zhou, Jianmei Liu, Shuai Wu, Wanran Li, Yun Zheng

**Affiliations:** ^1^ Department of Endocrinology and Metabolism, The First People’s Hospital of Yunnan Province, The Affiliated Hospital of Kunming University of Science and Technology, Kunming, China; ^2^ State Key Laboratory of Primate Biomedical Research, Institute of Primate Translational Medicine, Kunming University of Science and Technology, Kunming, China; ^3^ College of Horticulture and Landscape, Yunnan Agricultural University, Kunming, China

**Keywords:** growth hormone deficiency (GHD), *ZNF462*, Weiss–Kruszka syndrome (WSKA), mutation, case report

## Abstract

Weiss–Kruszka syndrome (WSKA) is a rare disease most often caused by mutations in the *ZNF462* gene. To screen for hereditary diseases, exons from the patient’s genome were sequenced. Genomic PCR experiments followed by Sanger sequencing were used to confirm the mutated genomic regions in the patient and his parents. We report a new mutation site, a heterozygous mutation (NM_021224.6:c.6311dup) in *ZNF462* in a male patient of 8 years old. The mutation in the *ZNF462* gene caused WSKA. This patient is the first case with WSKA characterized by attention-deficit hyperactivity disorder and complete growth hormone deficiency without pituitary lesions. Our results suggest that the heterozygous mutation in *ZNF462* is the direct cause of WSKA in this patient. Mutations in other genes interacting with *ZNF462* result in similar symptoms of WSKA. Furthermore, *ZNF462* and its interacting proteins ASXL2 and VPS13B may form a protein complex that is important for normal development but awaits more studies to reveal its detailed functions.

## 1 Introduction

Weiss–Kruszka syndrome (WSKA) is usually characterized by mild overall developmental delays and common craniofacial abnormalities, according to the OMIM database (Amberger et al., 2019). It is a rare genetic disorder, usually associated with *ZNF462* gene dysfunction, with reported autosomal dominant inheritance (Kruszka, 1993).

The *ZNF462* gene is located on chromosome 9p31.2 and encodes the protein ZNF462, which belongs to the C2H2 zinc finger protein family (C2H2-ZNF) (Al-Naama et al., 2020). The function of ZNF462 is unclear, but studies have shown that it plays a key role in early embryonic development and neuronal differentiation (Laurent et al., 2009; Massé et al., 2011). ZNF462 is expressed in a graded pattern in the mouse cerebral cortex, with the strongest expression in the marginal zone, cortical plate, and subventricular zone of the cortical layer (Chang et al., 2007). Homozygous *ZNF462* knockout (*ZNF462* ^−/−^) has been shown to be fatal in mice, while heterozygous (*ZNF462* ^+/−^) mice showed anxiety-like stunting behavior, low brain weight, and over-self-grooming; anxiety symptoms and over-self-grooming behavior were alleviated after imipramine treatment (Wang et al., 2017).

At present, 30 cases of WSKA associated with *ZNF462* gene mutations have been reported. Among them, 28 studies did not investigate other genes but only the effect of *ZNF462* mutation on WSKA disease. One study investigated the chromosomal rearrangement of *ASXL2* and *KIAA1803* in WSKA patients (Ramocki et al., 2003), and the other study reported that disrupted *KLF12* and *ZNF462* incurred similar WSKA symptoms (Cosemans et al., 2018). Our study reports a new mutation of *ZNF462* that differs from those previously reported. We also discuss the effects of genes cooperating with *ZNF462* on disease.

## 2 Case description

### 2.1 Symptoms and physical and routine examination

A boy aged 8 years and 6 months came to the Affiliated Hospital at Kunming University of Science and Technology (the First People’s Hospital of Yunnan Province) for evaluation of growth retardation. His parents are non-consanguineous. The proband was born *via* spontaneous vaginal delivery at 40 weeks, with birth length 50.0 cm (27.3rd) and weight 2.8 kg (5.3rd). He has no brothers or sisters. The heights of his father and mother are 167.0 cm and 157.0 cm, respectively.

At the first visit, the height of the proband was 126.5 cm (14.0th), weight 34.1 kg, and BMI 21.3 kg/m^2^ (obese). He had a metopic ridge, arched eyebrows, bilateral ptosis, epicanthal folds, down-slanting palpebral fissures, a short upturned nose with a bulbous tip, and a marked cupid bow (Supplementary Figure S1). The ears were low-set, but his hearing was normal. There was no significant difference between the patient and his peers in speech or motor development, and no feeding difficulties or snoring problems. The proband has no other congenital abnormalities except cryptorchidism. There is no history of ptosis, dwarfism, or intellectual disability in other family members.

Upon the proband’s routine inspection, his liver, kidneys, electrolytes, blood fat, and thyroid were normal. Testing revealed a FSH of 1.45 mIU/ml (3.5–12.5 mIU/ml), LH < 0.100 pmol/L (1.24–8.62 pmol/L), E2 42.35 pmol/L (20–75 pmol/L), Prog 0.452 nmol/L, PRL 10.68 ng/ml, and T < 0.087 pmol/L. Serum 25 hydroxyvitamin D was 15.07 ng/ml (>30 ng/ml), and PTH was 49.1 pg/ml (14–72 pg/ml). The serum IGF-1 and IGFBP-3 levels were 174 ng/ml (64.00–358.00 ng/ml) and 4.09 ng/ml (1.6–6.5 ng/ml), respectively. A growth hormone excitation test with levodopa combined with arginine was conducted, and it produced a peak of 1.35 ng/ml, indicating complete growth hormone deficiency (GHD).

His bone age was 1.6 years older than his chronological age. According to his bone age and other elements, his final height should be 156.8 cm (<3rd percentile of the normal heights of peers in the same area). Cranial magnetic resonance imaging (MRI) showed demyelinating changes in both parietal lobes, but the structures of the corpus callosum and pituitary gland were intact. Sleep monitoring indicated obstructive sleep apnea (OSA). Bone mineral density (BMD) was normal (Z:1.4). Fundus examination and echocardiography were normal.

The patient was evaluated by Wechsler intelligence assessment and Chinese children cognitive system. The comprehensive score of cognitive ability was 78 points that was lower than the average of his peers, and his cognitive level was located at the lowest 7.0% level of his peers. The results of the patient’s comprehensive attention test showed that the patient had attention deficit hyperactivity disorder (ADHD).

### 2.2 Materials and methods

#### 2.2.1 Sample information and sequencing profiles obtained

Peripheral blood samples from the patient were collected at the First People’s Hospital of Yunnan Province. Whole-exon sequencing was performed by Beijing Golden Gene Technology Co., Ltd. The obtained exon sequencing has been stored in the NCBI SRA database under the accession number SRR18969535.

#### 2.2.2 Analysis of the exon sequencing profile to identify genomic mutations

FastQC (Andrews, 2010) was used to check the sequencing quality of the obtained whole-exon sequencing data, and clean reads were obtained by fastp (Chen et al., 2018). BWA (v0.7.5a-r405) (Richard & Heng, 2009) was used to align the clean reads of the sequencing file to human genome sequences (hg38) downloaded from the UCSC Genome Browser (Kent et al., 2002). The resulting SAM file was converted to a BAM file using SAMtools (Li et al., 2009). Next, the BAM file was sorted with SAMtools and used to identify mutations with GATK (v4.2.1.0) (McKenna et al., 2010). Briefly, the MarkDuplicates program was used to remove PCR duplicates; BaseRecalibrator and ApplyBQSR were used to recalibrate the base quality score; and then, Mutect2 was used to identify mutations in exons and to produce a VCF file of the identified mutations. Then, ANNOVAR (v20200607) (Wang et al., 2010) was used to annotate the VCF file to obtain the gene mutations.

#### 2.2.3 Verification of the new mutation in *ZNF462*

Whole-blood samples from the patient and his parents were collected. Then, the DNA samples were extracted and sequenced by Sanger sequencing. The obtained sequences were aligned to the human genomic sequence (hg38) with BLASTN (Altschul et al., 1990). A heterozygous insertion of adenosine was found in the seventh exon of the *ZNF462* gene by manual examination with IGV (Thorvaldsdottir et al., 2013). The protein–protein interaction network of ZNF462 was studied using STRING (v11.5) (Szklarczyk et al., 2021).

The reported mutations in *ZNF462* were manually retrieved from the literature. Then, 30 reported mutations in *ZNF462* and the new mutation in *ZNF462* identified in this study were visualized with trackViewer (v1.6.1) (Ou & Zhu, 2019).

#### 2.2.4 GO term analysis for ZNF462 and its interacting proteins

Gene Ontology (GO) enrichment analysis of ZNF462 and its interacting proteins was performed with KOBAS (v3.0) (Bu et al., 2021). The obtained GO terms were grouped into three major categories: molecular function, cellular component, and biological process. The top 10 GO terms with the smallest multiple test-corrected *p*-values in these three categories were chosen for visualization.

### 2.3 Results

#### 2.3.1 Characterizing the genomic mutations of the patient

**FIGURE 1:**
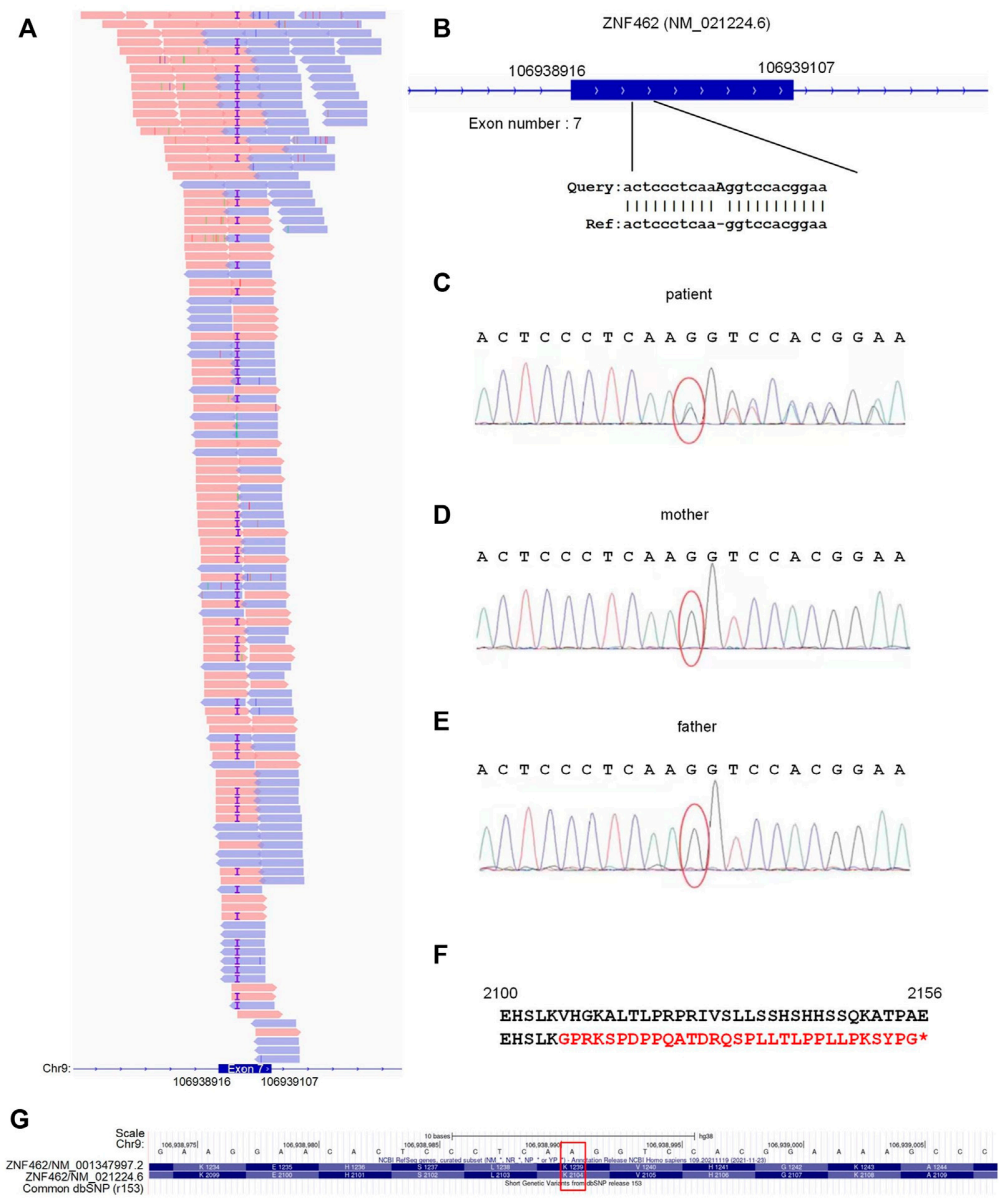
Identified mutation locus of *ZNF462* in the patient. **(A)** Locus of the adenosine insertion in *ZNF462* and the exome sequencing reads at the locus. The reads marked with purple “I”s are those with the adenosine insertions. **(B)** Insertion of adenosine occurs in the seventh exon of *ZNF462* (NM_021224.6). **(C–E)** Sanger sequencing results of the patient and his parents. Red ovals indicate the insertion site of adenosine. **(F)** Amino acid sequences of *ZNF462* without (up) and with the adenosine insertion (down). In the mutated *ZNF462* amino acid sequence, the adenosine insertion causes a variation from Val to Gly and different subsequent amino acids, and introduces premature translation terminations. **(G)** When compared with the dbSNP (r153) in the UCSC Genome Browser, the identified insertion site of adenosine was not a known SNP, as indicated by the red rectangle.

To obtain a molecular diagnosis, exon sequencing was performed for the patient. Figure 1A shows the high-throughput sequencing reads for the seventh exon of the patient’s *ZNF462* gene, where 54% of these sequences carried the A insertion (or duplication), suggesting a heterozygous mutation at the A insertion site in the patient. Using GATK to analyze the exon sequencing data revealed an insertion of adenosine at the seventh exon of the *ZNF462* gene [NM_021224.6: c.6311dup; p. (Val2105GlyfsTer32)] (Figure 1B). Then, we sequenced the same genomic regions in the patient and his parents with Sanger sequencing (Figures 1C–E). In the Sanger sequencing results for the patient, there were continuous secondary peaks from the insertion site of adenosine (red oval in Figure 1C), suggesting two different sequences for the heterozygous insertion site of adenosine. However, as shown in Figures 1D,E, the Sanger sequencing of the mother and father clearly indicated no mutations. As shown in Figure 1F, due to a code shift introduced by the additional adenosine, valine (Val) at the 2105th amino acid was transformed into glycine (Gly); the subsequent 32 amino acids were encoded incorrectly and terminated prematurely. Therefore, the heterozygous A insertion may lead to a half-loss of functional ZNF462 protein in the patient. The mutation (NM_021224.6:c.6311dup) was not a reported SNP after being compared to dbSNP (v153) Figure 1G.

#### 2.3.2 The protein–protein interaction network of ZNF462

**FIGURE 2:**
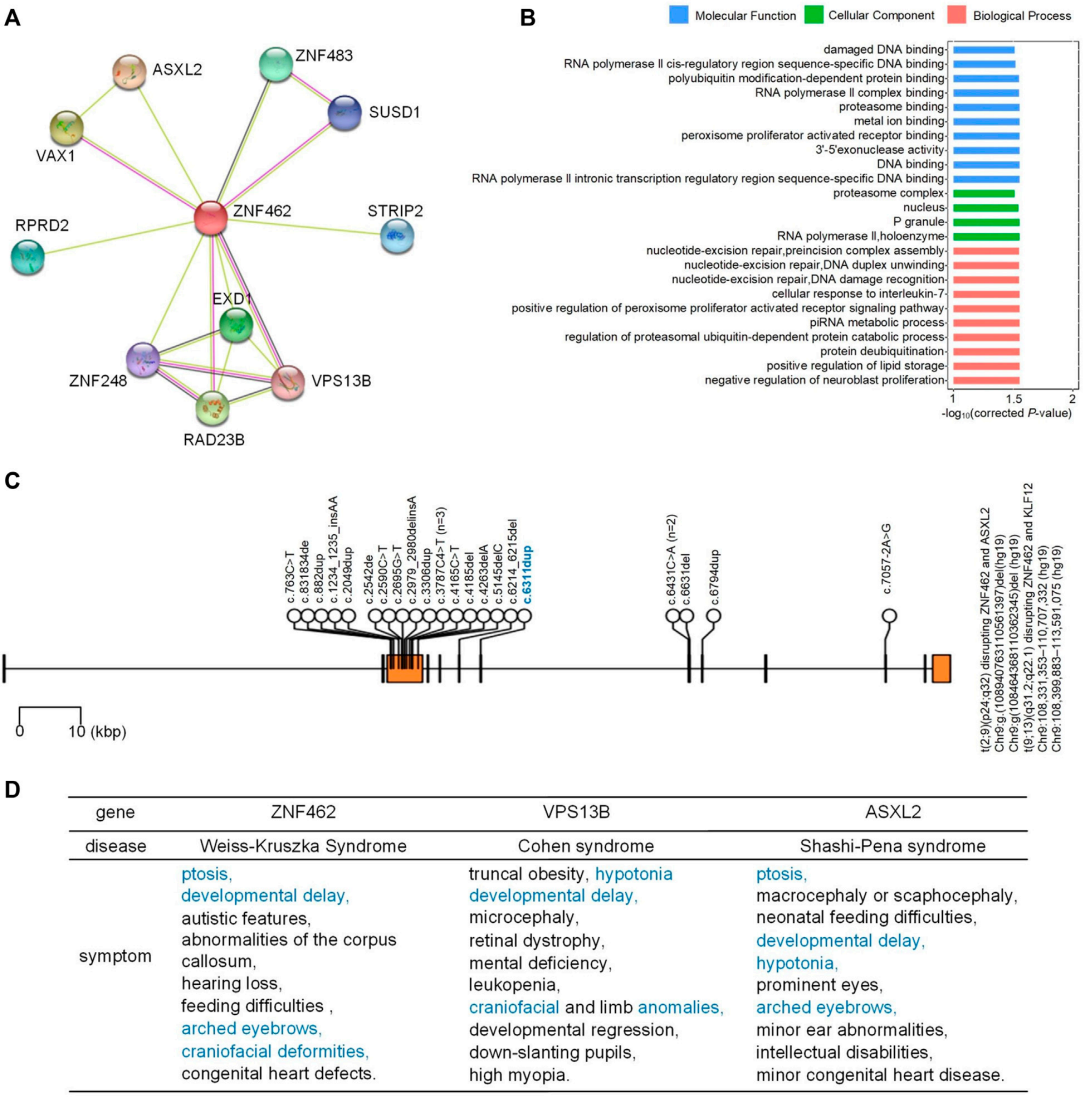
Proteins interacting with ZNF462 and mutations in ZNF462. **(A)** Proteins interacting with ZNF462. STRING (v 11.5) was used to prepare the figure. **(B)** Enriched GO terms for genes in **(A)**. Only top GO terms were shown, and details are provided in Materials and Methods. **(C)** Reported mutations in *ZMF462* that are related to WSKA. The blue mutation is the one newly identified in this study. See Supplementary Table S1 for details. **(D)** Diseases and symptoms caused by genetic mutations in *ZNF462*, *VPS13B*, and *ASXL2*. Common symptoms of at least two diseases are shown in blue.

We next examined the interacting proteins of ZNF462 with STRING (v11.5) (Talisetti et al., 2003), as shown in Figure 2A. To determine the function of the ZNF462 protein and its interacting proteins, GO enrichment analysis was used with KOBAS. The top GO terms for these genes are “polyubiquitin modification-dependent protein binding,” “RNA polymerase II complex binding,” “proteasome binding,” “metal ion-binding,” “peroxisome proliferator-activated receptor binding,” “3′-5′ exonuclease activity,” “DNA binding,” and “RNA polymerase II intronic transcription regulatory region sequence-specific DNA binding,” as shown in Figure 2B.

We also manually retrieved the previously reported *ZNF462* mutations related to WSKA, then visualized them with the mutation newly identified in this study (Figure 2C). Most reported mutations are located in the third exon of *ZNF462* (details in Supplementary Table S1). The new mutation identified in this study is located in the seventh exon of the *ZNF462* gene.

Among the proteins interacting with ZNF462, the *ASXL2* gene encodes a member of the epigenetic regulator family that binds various histone modification enzymes and participates in the assembly of transcription factors at specific genomic sites (Lai & Wang, 2013). Recent studies have found that pathogenic mutations in the *ASXL2* gene could lead to Shashi–Pena syndrome, which is characterized by facial abnormalities and developmental delays (Shashi et al., 2016) (Figure 2D). *ASXL2*-deficient mice showed weight loss, enlarged hearts, and skeletal abnormalities (Baskind et al., 2009). Chromosome translocation t (2; 9) leads to a fused transcript of *ASXL2* and *KIAA1803*, resulting in a complex phenotype of the corpus callosum, ocular colobomas, and periventricular ectopic dysplasia (Ramocki et al., 2003). Weiss et al. (2017) have suggested that *ZNF462* and *ASXL2* might work together in the WSAK phenotype.

VPS13B is another protein interacting with ZNF462 (Figure 2A). Mutations in the *VPS13B* gene have been associated with Cohen’s syndrome, a rare autosomal recessive syndrome associated with a variety of clinical manifestations including growth retardation, hypotonia, joint hyperactivity, microcephaly, intellectual impairment, and craniofacial and limb abnormalities (Cohen et al., 1973) (Figure 2D). The VPS13 protein family is involved in vesicular transportation and membrane events (Kolehmainen et al., 2004). To date, more than 150 VPS13B mutations have been reported in more than 200 patients with Cohen syndrome (Momtazmanesh et al., 2020).

VAX1 interacts with both ZNF462 and ASXL2 (Figure 2A). Heterozygous deletion of *Vax1* leads to infertility in mice and irregular estrous cycles in female mice (Hoffmann et al., 2014).

These results suggest that mutations in other genes interacting with *ZNF462* (Figure 2A) may result in symptoms similar to WSKA (Figure 2D). We hypothesize that ZNF462 and its interacting proteins may form a protein complex that is important for normal development and which awaits more studies to reveal its detailed functions.

## 3 Diagnosis and treatment

In summary, the patient was diagnosed with WSKA syndrome, GHD, dwarfism, intellectual disability, and ADHD. The proband had bilateral cryptorchidism and underwent surgery 3 months later. To promote his height growth, the proband received 3.4U/day (0.1 U/kg/day) subcutaneous injections of recombinant human growth hormone (rhGH). However, the IGF of this patient significantly increased and exceeded the normal range after normal and even lower usage of rhGH (Table 1). Considering the side effects of high IGF-1 levels, the patient discontinued rhGH treatment, and the IGF-1 dropped to the normal range. Finally, the patient had grown 6.5 cm in 7 months during the rhGH injections. After stopping the rhGH injections, the patient only grew 1 cm in 3 months. The patient was therefore given rhGH again, and his ADHD improved after using tomoxetine.

**TABLE 1:** Timeline with relevant data from the episode of care.

Time	Examination	Treatment	Diagnosis
2021.07 (8 years and 6 months)	Height: 126.5 cm; weight: 34.1 kg	Whole-exon sequencing	GHD, dwarfism, and bilateral cryptorchidism
	Peak of the growth hormone excitation test: 1.4 ng/ml		
	Bone age: 10 years and 1 month (method:TW3-RUS)		
	MRI: the pituitary gland and corpus callosum were normal		
	B ultrasound: bilateral cryptorchidism		
	IGF-1:17 4 .0 ng/ml (64.0–358.0 ng/ml)		
2021.08	Height: 126.5 cm; weight: 34.1 kg	Injection of rhGH 3.4 U/d	WSKA, GHD, dwarfism, and bilateral cryptorchidism
	Mutation in *ZNF462* (NM_021224.6:c.6311dup)	Cryptorchidism surgery	
2021.12	Height: 129.0 cm; weight: 40.2 kg	Injection of rhGH 3.0 U/d	
	IGF: 540.5 ng/ml (64.0–358.0 ng/ml)		
2022.01 (9 years)	Height: 130.0 cm; weight: 39.6 kg	Injection of rhGH 3.0 U/d	
	Glucose tolerance, liver and kidney function, and blood lipids were normal		
	IGF: 480.5 ng/ml (73.0–385.0 ng/ml)		
2022.02 (9 years and 1 month)	Height: 131.0 cm; weight: 39.6 kg	Injection of rhGH 2.5 U/d	WSKA, GHD, dwarfism, intellectual disability, and ADHD
	Bone age: 11 years and 1 month (method Rus-CHN)		
	Composite score intelligence quotient: 78		
	Comprehensive attention test: ADHD		
	IGF: 462.16 ng/ml (73.0–385.0 ng/ml)		
2022.04	Height 133.0 cm; weight: 40 kg	Withdrawal of rhGH	
	IGF: 460.22 ng/ml (73.0–385.0 ng/ml)		
2022.07 (9 years and 6 months)	Height 134.0 cm; weight: 40 kg	Injection of rhGH 4.5 U/d	
	Bone age: 11 years and 10 months		
	Serum sex hormone levels were normal		
	IGF: 171.95 ng/ml (73.0–385.0 ng/ml)		
Now	—	Injection of rhGH 4.5 U/d	

Remarks: The three bone age tests were all different from the same instrument; IGF-1, reference range: 8 years old (64.0–358.0 ng/ml); 9 years old (73.0–385.0 ng/ml).

## 4 Discussion

The patient showed growth retardation associated with GHD, but his level of IGF-1 and advanced bone age were inconsistent with the clinical symptoms of GHD. We speculate that the patient was IGF-1-insensitive and that a mutation in his *ZNF462* gene caused WSKA, a rare genetic disease often accompanied by growth retardation, craniofacial malformation, and/or corpus callosum dysplasia (González-Tarancón et al., 2020).

Since the first case of WSKA was reported in 2003 (Talisetti et al., 2003), only 30 patients have been reported. According to Table 2, most of them (74%) have some type of developmental delay, with speech delay being the most common, accounting for 42%, and motor delay being the second, noticed in 39% of patients. Almost half of patients have hypotonia, which is a contributor to motor delay. A few of them (16%) have intellectual disabilities. It cannot be ignored that 39% of patients have feeding problems.

**TABLE 2:** Common clinical manifestations and frequency of WSKA patients.

Item	Number	Frequency
Inheritance	—	—
*De novo*	23	74%
Paternally/maternally inherited	5	16%
Unknown	3	10%
ACC	7	23%
Metopic ridging/cranio synostosis	14	45%
Developmental delay	24	77%
Speech	13	42%
Motor	12	39%
Intellectual disability	5	16%
ASD	8	26%
Hypotonia	15	48%
Ptosis	27	87%
Down-slanting palpebral fissures	18	58%
Arched eyebrows	16	52%
Epicanthal folds	13	42%
Short upturned nose with bulbous tip	14	45%
Exaggerated cupid bow/wide philtrum	18	58%
Feeding issues	12	39%
Congenital heart disease	7	23%
Limb anomalies	6	19%
Ears	16	52%
Other	—	—
GHD	2	7%
Microrchidia/cryptorchid	3	10%
Attention disorder	3	10%
OSA	3	10%

Abbreviations: ACC: corpus callosum dysgenesis; ASD: autism spectrum disorder; GHD: growth hormone deficiency; OSA: obstructive sleep apnea.

We found that common craniofacial features include ptosis (87%), down-slanted palpebral fissures (58%), exaggerated cupid bow/wide philtrum (58%), arched eyebrows (52%), ear malformation/hearing loss (52%), short upturned nose with bulbous tip (45%), metopic ridging/craniosynostosis (45%), and epicanthal folds (42%). Corpus callosum dysgenesis is the most frequent manifestation of craniocerebral dysplasia, which is also considered to be a typical manifestation of WSKA but occurs in only 23% of patients.

Twenty-six percent of patients have autism spectrum disorder (ASD), which is consistent with the idea that ZNF462 disorder is an independent risk factor for autism (Krumm et al., 2015). However, this patient shows ADHD, which may be related to the demyelination of white matter in the parietal lobe (Silk et al., 2009). However, patients 5, 8, and 28 also have attention disorder. Furthermore, ASD, attention deficit, and ADHD have high rates of co-occurrence (Ohta et al., 2020). Therefore, the patient’s ADHD might be caused by his *ZNF462* genetic mutation.

Seventy-four percent of WSKA patients have developmental delay, but only 12 patients had their height described. The patient identified in this study has WSKA with GHD, which is similar to the patient reported by Park et al. (2021). However, Park reported a patient with low bone age, empty sella syndrome, adenohypophysis dysfunction, and delayed puberty, while our patient’s pituitary structure is intact (Park et al., 2021). It may be that the *ZNF462* mutation is responsible for the lack of growth hormone in WSKA patients, rather than the structural and functional dysfunction of the pituitary gland.

The patient identified in this study has a mutation at 9p31.2 on the *ZNF462* gene and shows GHD and short stature. Meanwhile, he presented with ptosis, arched eyebrows, down-slanting palpebral fissures, a short upturned nose with a bulbous tip, a marked cupid bow, and ADHD. This is the first report of a *ZNF462* gene mutation in patients with GHD but no pituitary lesions. It also provides new insights into endocrine symptoms related to dwarfism. When patients present with GHD and these craniofacial features, we should suspect WSKA.

## Data Availability

The data presented in the study are deposited in the NCBI SRA repository, accession number SRR18969535.
