# Supplementary material for: Case report: A heterozygous mutation in ZNF462 leads to growth hormone deficiency
Source: Front Genet. 2022 Dec 7;13:1015021. doi: 10.3389/fgene.2022.1015021 (PMC9770794; doi:10.3389/fgene.2022.1015021)
Supplement: Supplementary file 1 [file Table1.docx]

Supplementary Material

# Supplementary Table

Supplement Table S1 Phenotype characteristics of 31 WSKA patients.

| Patients | 1 | 2^a^ | 3^b^(proband) |
| --- | --- | --- | --- |
| Sex | Male | Female | Female |
| Age | 8 years and 7 months | 5 years | 2 years |
| ZNF462 variant | c.6311dup  p.V2105Gfs*32 | t(2;9)(p24;q32)  disrupting KIAA1803 and ASXL2 | c.3787C4﹥T  p.(Arg1263*) |
| Inheritance | De novo | De novo | Paternally inherited |
| Reported height | 126.5 cm (14th) | 99 cm (5th) | 70 cm (75th) |
| Cranial MRI | Demyelinate | ACC/dilated ventricles | ACC/dilated ventricles |
| Developmental delay | + | Speech/ motor | ﹣ |
| Intellectual disability | ﹢ | ﹢ | ﹣ |
| ASD | ﹣ | ﹢ | ﹣ |
| Hypotonia | ﹣ | ﹢ | ﹣ |
| Metopic ridge/Cranio synostosis | ﹢ | ﹢ | ﹢ |
| Ptosis | ﹢ | ﹢ | ﹢ |
| Down slanting palpebral fissures | ﹢ | ﹢ | ﹢ |
| Arched eyebrows | ﹢ | ﹢ | ﹢ |
| Epicanthal folds | ﹢ | ﹣ | ﹣ |
| Short up turned nose with bulbous tip | ﹢ | ﹢ | ﹢ |
| Exaggerated cupid bow/ wide philtrum | ﹢ | ﹢ | ﹢ |
| Feeding issues | ﹣ | ﹢ | ﹣ |
| Congenital heart disease | ﹣ | VSD/ left ventricular hypertrophy | ﹣ |
| Limb anomalies | ﹣ | Single palmar crease/hypoplastic finger nails | ﹣ |
| Ears | Low-set ears | Low-set ears/hearing loss | ﹣ |
| Other | GHD /cryptorchid/  overweight/ADHD/OSA | OSA / hydronephrosis / chorioretinal colobomas | Congenital capillary malformation |

| Patients | 4 ^b^(sister) | 5 ^b^(father) | 6 ^b^ | 7 ^b^ | 8 ^b^ |
| --- | --- | --- | --- | --- | --- |
| Sex | Female | Male | Male | Male | Male |
| Age | 4 years | 34 years | 2 years | 32 months | 5 years |
| ZNF462 variant | c.3787C4﹥T  p.(Arg1263*) | c.3787C4﹥T  p.(Arg1263*) | c.2979_2980delinsA  p.(Val994Trpfs*147) | c.4263delA  p.(Glu1422Serfs*6) | Chr9:g.(108940763110561397)del(hg19) |
| Inheritance | Paternally inherited | Maternally inherited | De novo | De novo | De novo |
| Reported height | 66 cm (50th) | 168 cm (10th) | 91 cm (27th) | 82.5 cm (34th) | 118 cm (97th) |
| Cranial MRI | ﹣ | ﹣ | ﹣ | ACC/dilated ventricles | ACC |
| Developmental delay | ﹣ | ﹣ | Speech | Speech/ motor | ﹣ |
| Intellectual disability | ﹣ | ﹣ | ﹣ | ﹣ | ﹣ |
| ASD | ﹣ | ﹣ | ﹢ | ﹢ | ﹣ |
| Hypotonia | ﹣ | ﹣ | ﹣ | ﹢ | ﹢ |
| Metopic ridge/Cranio synostosis | ﹢ | ﹢ | ﹢ | ﹢ | ﹣ |
| Ptosis | ﹢ | ﹢ | ﹢ | ﹢ | ﹢ |
| Down slanting palpebral fissures | ﹢ | ﹣ | ﹢ | ﹢ | ﹢ |
| Arched eyebrows | ﹣ | ﹣ | ﹢ | ﹣ | ﹣ |
| Epicanthal folds | ﹢ | ﹣ | ﹢ | ﹢ | ﹢ |
| Short up turned nose with bulbous tip | ﹢ | ﹣ | ﹢ | ﹢ | ﹢ |
| Exaggerated cupid bow/ wide philtrum | ﹢ | ﹣ | ﹣ | ﹣ | ﹢ |
| Feeding issues | ﹣ | ﹣ | ﹣ | ﹣ | ﹣ |
| Congenital heart disease | ﹣ | ﹣ | ﹣ | D-TGA/PDA | ﹣ |
| Limb anomalies | ﹣ | ﹣ | Fifth finger clinodactyly/ Single palmar crease | ﹣ | ﹣ |
| Ears | ﹣ | ﹣ | Asymmetric ears | Right ear folded /the left cupped | ﹣ |
| Other | ﹣ | ﹣ | Small widely spaced teeth | Silent aspirations on swallow exam | Overweight |

| Patients | 9 ^b^ | 10 ^b^ | 11^c^ | 12^d^ |
| --- | --- | --- | --- | --- |
| Sex | Male | Male | Male | Male |
| Age | 12 years | 9 years | 24 years | 16 months |
| ZNF462 variant | Chr9:g(108464368110362345)del (hg19) | c.5145delC.  Tyr1716Thrfs*28 | t(9;13)(q31.2;q22.1)  disrupting ZNF462 and KLF12 | c.2590C>T  p.(Arg864*) |
| Inheritance | De novo | De novo | De novo | Maternally inherited |
| Reported height | / | 133.5 cm(25th) | 161 cm（<3rd） | / |
| Cranial MRI | Not tested | ﹣ | ACC | ﹣ |
| Developmental delay | Motor | Speech | Speech/ Motor | Speech/ motor |
| Intellectual disability | ﹢ | ﹣ | ﹢ | ﹣ |
| ASD | ﹢ | ﹢ | ﹢ | ﹣ |
| Hypotonia | ﹣ | ﹢ | ﹢ | ﹢ |
| Metopic ridge/Cranio synostosis | ﹣ | ﹣ | ﹢ | ﹣ |
| Ptosis | ﹣ | ﹢ | ﹢ | ﹢ |
| Down slanting palpebral fissures | ﹣ | ﹢ | ﹢ | ﹣ |
| Arched eyebrows | ﹣ | ﹣ | ﹢ | ﹢ |
| Epicanthal folds | ﹣ | ﹣ | ﹢ | ﹢ |
| Short up turned nose with bulbous tip | ﹣ | ﹢ | ﹣ | ﹣ |
| Exaggerated cupid bow/ wide philtrum | ﹢ | ﹣ | ﹢ | ﹢ |
| Feeding issues | ﹣ | ﹣ | ﹢ | ﹢ |
| Congenital heart disease | ﹣ | ﹣ | ﹣ | ﹣ |
| Limb anomalies | ﹣ | ﹣ | Small hands and feet/ Single palmar crease | Fifth finger clinodactyly |
| Ears | ﹣ | ﹣ | Low-set ears | Low-set ears |
| Other | Attention deficit disorder/obsessive compulsive disorder. | Umbilical hernia repair | Overweight | ﹣ |

| Patients | 13^d^ | 14 ^d^ | 15 ^d^ | 16 ^d^ | 17 ^d^ |
| --- | --- | --- | --- | --- | --- |
| Sex | Male | Male | Male | Female | Female |
| Age | 10 years | 6 years | 2 years 7 months | 14 years | 7 months |
| ZNF462 variant | c.2542del  p.(Cys848Valfs*66) | c.831_834del  p.(Arg277Serfs*26) | c.6214_6215del  p.(His2072Tyrfs*8) | c.763C > T  p.(Arg255*) | c.7057﹣2A > G |
| Inheritance | De novo | De novo | De novo | De novo | De novo |
| Reported height | / | / | / | / | / |
| Cranial MRI | ﹣ | ﹣ | Not tested | Not tested | ﹣ |
| Developmental delay | Speech/ motor | Speech/ motor | Speech | IEP | Early intervention |
| Intellectual disability | ﹣ | ﹣ | ﹣ | ﹣ | ﹣ |
| ASD | ﹢ | ﹣ | ﹣ | ﹣ | ﹣ |
| Hypotonia | ﹣ | ﹢ | ﹣ | ﹣ | ﹢ |
| Metopic ridging /cranio synostosis | ﹣ | ﹣ | ﹢ | ﹢ | ﹣ |
| Ptosis | ﹢ | ﹣ | ﹢ | ﹢ | ﹢ |
| Down slanting palpebral fissures | ﹢ | ﹣ | ﹣ | ﹣ | ﹢ |
| Arched eyebrows | ﹢ | ﹣ | ﹣ | ﹢ | ﹢ |
| Epicanthal folds | ﹢ | ﹣ | ﹢ | ﹢ | ﹢ |
| Short up turned nose with bulbous tip | ﹢ | ﹢ | ﹣ | ﹣ | ﹢ |
| Exaggerated cupid bow/ wide philtrum | ﹢ | ﹣ | ﹢ | ﹣ | ﹢ |
| Feeding issues | ﹢ | ﹢ | ﹢ | ﹣ | ﹢ |
| Congenital heart disease | ﹣ | VSD/bicuspid aortic valve | ﹣ | ﹣ | VSD |
| Limb anomalies |  |  |  | ﹣ | Prominent creases on hands and feet |
| Ears |  |  |  | Hearing loss | Horizontal crus helix |
| Other |  |  |  | Father had ptosis surgery | ﹣ |

| Patients | 18^d^ | 19 ^d^ | 20 ^d^ | 21 ^d^ | 22 ^d^ |
| --- | --- | --- | --- | --- | --- |
| Sex | Male | Male | Male | Male | Female |
| Age | 13 years | 2 years | 15 years | 8 years | 2 years and 5 months |
| ZNF462 variant | c.6794dup  p.(Tyr2265*) | c.882dup  p.(Ser295Glnfs*64) | c.4165C > T  p.(Gln1389*) | c.1234_1235insAA  p.(Ser412*) | c.6214_6215del  p.(His2072Tyrfs*8) |
| Inheritance | De novo | De novo | De novo | Unknown | De novo |
| Reported height | / | / | / | / | / |
| Cranial MRI | Not tested | ACC | Not tested | ﹣ | Not tested |
| Developmental delay | Cognitive impairment | Speech | Global | Speech/ motor /IEP | ﹣ |
| Intellectual disability | ﹣ | ﹣ | ﹣ | ﹣ | ﹣ |
| ASD | ﹢ | ﹣ | ﹣ | ﹣ | ﹣ |
| Hypotonia | ﹣ | ﹣ | ﹢ | ﹣ | ﹢ |
| Metopic ridging /cranio synostosis | ﹢ | ﹣ | ﹣ | ﹣ | ﹣ |
| Ptosis | ﹣ | ﹢ | ﹢ | ﹢ | ﹢ |
| Down slanting palpebral fissures | ﹣ | ﹢ | ﹢ | ﹣ | ﹣ |
| Arched eyebrows | ﹢ | ﹣ | ﹢ | ﹣ | ﹣ |
| Epicanthal folds | ﹣ | ﹣ | ﹣ | ﹣ | ﹣ |
| Short up turned nose with bulbous tip | ﹣ | ﹣ | ﹣ | ﹣ | ﹣ |
| Exaggerated cupid bow/ wide philtrum | ﹢ | ﹣ | ﹢ | ﹣ | ﹣ |
| Feeding issues | ﹣ | ﹣ | ﹢ | ﹢ | ﹢ |
| Congenital heart disease | ﹣ | ﹣ | ﹣ | ﹣ | ﹣ |
| Limb anomalies | ﹣ | ﹣ | ﹣ | ﹣ | ﹣ |
| Ears | Prominent ears/ear pits/hearing loss | ﹣ | Low-set ears | Mildly cupped ears | ﹣ |
| Other | ﹣ | ﹣ | ﹣ | ﹣ | ﹣ |

| Patients | 23 ^d^ | 24 ^d^ | 25^d^ | 26^e^ |
| --- | --- | --- | --- | --- |
| Sex | Male | Male | Female | Male |
| Age | 9 months | 8 years and 7 months | 8 years | 3 years and 4months |
| ZNF462 variant | c.2049dup  p.(Pro684Serfs*14) | c.6631del  p.(Arg2211Glyfs*59) | c.2695G >T  p.(Glu899*) | c.3306dup  p.(Gln1103Thrfs*10) |
| Inheritance | De novo | De novo | Mother negative; father unknown | De novo |
| Reported height | / | / | / | 91.7 cm (4th) |
| Cranial MRI | ﹣ | Not tested | ﹣ | Not tested |
| Developmental delay | Motor | ﹣ | ﹣ | ﹣ |
| Intellectual disability | ﹣ | ﹣ | Cognitive impairment | Speech/ motor |
| ASD | ﹣ | ﹣ | ﹣ | ﹣ |
| Hypotonia | ﹢ | ﹣ | ﹢ | ﹣ |
| Metopic ridging /cranio synostosis | ﹣ | ﹣ | ﹣ | ﹢ |
| Ptosis | ﹢ | ﹢ | ﹣ | ﹢ |
| Down slanting palpebral fissures | ﹢ | ﹢ | ﹣ | ﹢ |
| Arched eyebrows | ﹢ | ﹣ | ﹢ | ﹢ |
| Epicanthal folds | ﹢ | ﹣ | ﹣ | ﹢ |
| Short up turned nose with bulbous tip | ﹢ | ﹢ | ﹣ | ﹣ |
| Exaggerated cupid bow/ wide philtrum | ﹢ | ﹢ | ﹣ | ﹢ |
| Feeding issues | ﹣ | ﹣ | ﹢ | ﹢ |
| Congenital heart disease | ﹣ | ﹣ | ﹣ | Ventricular septal hypertrophy |
| Limb anomalies | ﹣ | Fifth finger  clinodactyly | ﹣ | ﹣ |
| Ears | ﹣ | ﹣ | ﹣ | ﹣ |
| Other | ﹣ | ﹣ | ﹣ | Iron deficiency microcytic anemia/ OSA/ kyphosis |

| Patients | 27^f^ | 28^g^ | 29^h^ | 30^i^ (proband) | 31^i^(father) |
| --- | --- | --- | --- | --- | --- |
| Sex | Male | Male | Female | Male | Male |
| Age | 16 years | 13 years and 6 months | 4years | 8 months | 31 years |
| ZNF462 variant | c.4185del  p.(Met1396Ter) | Chr9:108,331,353–  110,707,332(hg19) | Chr9:108,399,883–113,591,075, hg19 | c.6431C > A  p.(Ser2144) | c.6431C > A  p.(Ser2144) |
| Inheritance | De novo | De novo | De novo | Paternally inherited | De novo |
| Reported height | 152.8 cm (<3rd) | / | / | / | / |
| Cranial MRI | ACC/ ESS | No olfactory bulbs | ﹣ | ﹣ | Not tested |
| Developmental delay | ﹢ | ﹢ | ﹢ | ﹣ | - |
| Intellectual disability | Speech | Global | Motor | Motor | - |
| ASD | ﹢ | ﹣ | ﹣ | ﹣ | - |
| Hypotonia | ﹣ | ﹣ | ﹣ | ﹣ | - |
| Metopic ridging /cranio synostosis | ﹢ | ﹣ | ﹣ | ﹢ | - |
| Ptosis | ptosis surgery | ﹢ | ﹢ | ﹢ | ptosis surgery |
| Down slanting palpebral fissures | ﹢ | ﹣ | ﹢ | ﹣ | - |
| Arched eyebrows | ﹢ | ﹣ | ﹢ | ﹣ | - |
| Epicanthal folds | ﹣ | ﹣ | ﹣ | ﹣ | - |
| Short up turned nose with bulbous tip | ﹢ | ﹣ | ﹣ | ﹣ | - |
| Exaggerated cupid bow/ wide philtrum | ﹢ | ﹣ | ﹢ | ﹣ | - |
| Feeding issues | ﹣ | ﹣ | ﹣ | ﹣ | - |
| Congenital heart disease | ﹣ | VSD（closed spontaneously） | ﹣ | PDA | / |
| Limb anomalies | ﹣ | ﹣ | ﹣ |  | - |
| Ears | ﹣ | Low-set /hearing loss | ﹣ | Hearing loss | Hearing loss |
| Other | GHD/ microrchidia | KS/ delayed puberty / microrchidia / attention deficit disorder | Acute lymphoblastic leukemia | Asymmetric face | - |

Abbreviation: **ACC**: Corpus callosum dysgenesis; **ADHD**: Attention deficit hyperactivity disorder; **ASD**: Autistic spectrum disorder; **ESS**: Empty sella syndrome; **GHD**: Growth hormone deficiency; **MRI**: Magnetic resonance imaging; **OSA**: Obstructive sleep apnoea: **WSKA**: Weiss-Kruszka syndrome.

**Patients:** Patient 1: The Patient of this study; Patient 2^a^: Ramocki et al. (2003); Patient 3-10^b^: Weiss et al. (2017); Patient 11^c^ : Cosemans et al. (2018); Patient 12-25^d^ Kruszka et al. (2019); Patient 26^e^: González﹣Tarancón et al. (2020); Patient 27^f^: Iivonen et al. (2021); Patient 28^g^: Park et al. (2021); Patient 29^h^: Pellino et al. (2022); and Patient 30-31^i^: Zhao et al. (2022).

# Supplementary Figure


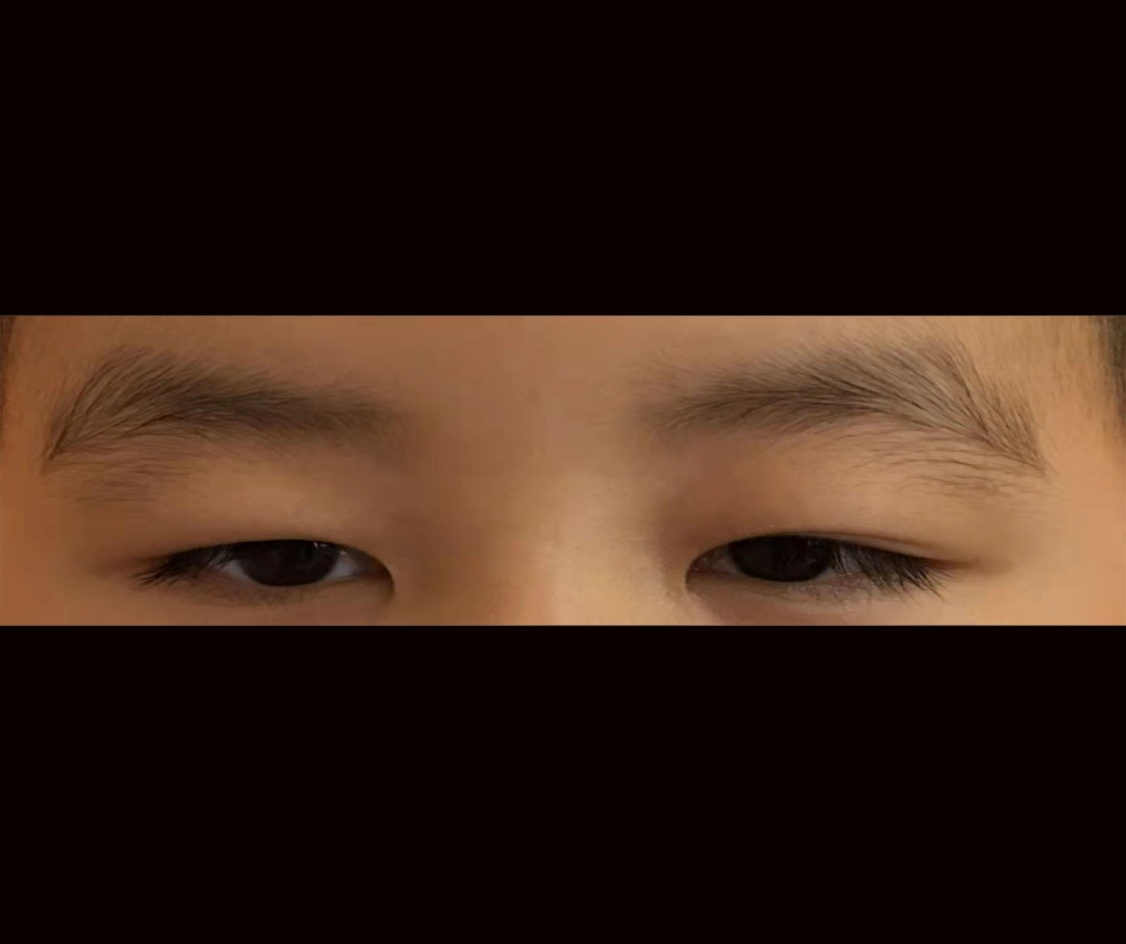


**Supplementary Figure S1.** The patient has arched eyebrows, ptosis, epicanthal folds and down slanting palpebral fissures. The figure is published with the consent of the patient and his parents.

**Supplementary References**

Iivonen, A. P., Kärkinen, J., Yellapragada, V., Sidoroff, V., Almusa, H., Vaaralahti, K., & Raivio, T. (2021). Kallmann syndrome in a patient with Weiss-Kruszka syndrome and a de novo deletion in 9q31.2. European journal of endocrinology, 185:1, 57–66. doi: 10.1530/EJE-20-1387

Kruszka, P., Hu, T., Hong, S., Signer, R., Cogné, B., Isidor, B., Mazzola, S. E., Giltay, J. C., van Gassen, K. L. I., England, E. M., Pais, L., Ockeloen, C. W., Sanchez-Lara, P. A., Kinning, E., Adams, D. J., Treat, K., Torres-Martinez, W., Bedeschi, M. F., Iascone, M., Blaney, S., … Muenke, M. (2019). Phenotype delineation of ZNF462 related syndrome. American journal of medical genetics. Part A, 179:10, 2075–2082. doi: 10.1002/ajmg.a.61306

Pellino, G., Chiasso, L., Fiori, G., Mazzone, S., Zama, D., Cordelli, D. M., & Russo, A. (2022). Acute lymphoblastic leukemia in a child with Weiss-Kruszka syndrome: Casual or causal association? European journal of medical genetics, 65:4, 104457. doi: 10.1016/j.ejmg.2022.104457

Zhao, S., Miao, C., Wang, X., Lu, Y., Liu, H., & Zhang, X. (2022). A Nonsense Variant of ZNF462 Gene Associated With Weiss-Kruszka Syndrome-Like Manifestations: A Case Study and Literature Review. Frontiers in genetics, 13, 781832. doi: 10.3389/fgene.2022.781832
